# Risk of death following admission to a UK hospital with diabetic ketoacidosis

**DOI:** 10.1007/s00125-016-4034-0

**Published:** 2016-07-11

**Authors:** Fraser W. Gibb, Wei Leng Teoh, Joanne Graham, K. Ann Lockman

**Affiliations:** 1 Edinburgh Centre for Endocrinology & Diabetes, Royal Infirmary of Edinburgh, Little France Crescent, Edinburgh, EH16 4SA UK; 2 Acute Medical Unit, Royal Infirmary of Edinburgh, Edinburgh, UK

**Keywords:** Deprivation, Diabetes, Diabetic ketoacidosis, HbA1c, Mortality, Type 1 diabetes

## Abstract

**Aims/hypothesis:**

The aim of this study was to assess the risk of death during hospital admission for diabetic ketoacidosis (DKA) and, subsequently, following discharge. In addition, we aimed to characterise the risk factors for multiple presentations with DKA.

**Methods:**

We conducted a retrospective cohort study of all DKA admissions between 2007 and 2012 at a university teaching hospital. All patients with type 1 diabetes who were admitted with DKA (628 admissions of 298 individuals) were identified by discharge coding. Clinical, biochemical and mortality data were obtained from electronic patient records and national databases. Follow-up continued until the end of 2014.

**Results:**

Compared with patients with a single DKA admission, those with recurrent DKA (more than five episodes) were diagnosed with diabetes at an earlier age (median 14 [interquartile range 9–23] vs 24 [16–34] years, *p* < 0.001), had higher levels of social deprivation (*p* = 0.005) and higher HbA_1c_ values (103 [89–108] vs 79 [66–96] mmol/mol; 11.6% [10.3–12.0%] vs 9.4% [8.2–10.9%], *p* < 0.001), and tended to be younger (25 [22–36] vs 31 [23–42] years, *p* = 0.079). Antidepressant use was greater in those with recurrent DKA compared with those with a single episode (47.5% vs 12.6%, *p* = 0.001). The inpatient DKA mortality rate was no greater than 0.16%. A single episode of DKA was associated with a 5.2% risk of death (4.1 [2.8–6.0] years of follow-up) compared with 23.4% in those with recurrent DKA admissions (2.4 [2.0–3.8] years of follow-up) (HR 6.18, *p* = 0.001).

**Conclusions/interpretation:**

Recurrent DKA is associated with substantial mortality, particularly among young, socially disadvantaged adults with very high HbA_1c_ levels.

**Electronic supplementary material:**

The online version of this article (doi:10.1007/s00125-016-4034-0) contains peer-reviewed but unedited supplementary material, which is available to authorised users.

## Introduction

Despite incremental improvements in mortality associated with diabetic ketoacidosis (DKA), 38% of deaths from hyperglycaemic crises occur at home in young adults. In the USA, there was very little change in the at-home mortality rate between 1985 and 2002 [1]. However, declining mortality rates from hyperglycaemic crises have been observed over a similar timescale [1]. Recent estimates of inpatient DKA mortality have varied between <1% [2] and up to 1.8% [3]. Type 1 diabetes continues to be associated with premature death and, in Scottish adults younger than 50 years, DKA has been identified as the single biggest contributor to loss of life (29.4% in men and 21.7% in women) [4]. Hospital admission for DKA is associated with social deprivation [5, 6], high HbA_1c_ levels [5–8], reduced concordance with insulin therapy [9] and female sex [5]. Although several case series of patients with ‘brittle diabetes’ (characterised at least in part by recurrent DKA) have been published [10–12], a robust definition of ‘brittle diabetes’ does not exist and no systematic assessment of mortality risk, in the context of recurrent DKA, has been conducted. We therefore sought to examine factors associated with recurrent admissions for DKA, to assess the risk of mortality during inpatient admissions for DKA and, for the first time, to determine the risk of mortality in the years following hospital discharge.

## Methods

This was a retrospective cohort study including all people with type 1 diabetes admitted with DKA to the Royal Infirmary of Edinburgh, a large university teaching hospital, over a 6-year period (2007–2012). Patients were identified from electronic discharge coding (which included patients who died during an admission). This was cross-checked against comprehensive mortality data from the national Scottish Care Information–Diabetes Collaboration (SCI-Diabetes) database (a dynamic clinical information system containing >99% of diagnosed diabetes cases in Scotland), over the same period, to ensure any DKA deaths were not missed in the original data collection. Electronic patient records and the SCI-Diabetes database were interrogated to record the date of diabetes diagnosis, social deprivation status (using the Scottish Index of Multiple Deprivation [SIMD], which comprises 6505 ‘datazones’, where ‘1’ is the most deprived and ‘6505’ is the least deprived) [13], the most recent HbA_1c_ level, length of inpatient hospital stay, laboratory variables on admission (including glucose, hydrogen ion, lactate, urea, creatinine and white cell count) and diabetes complications (micro- and macrovascular).

The complete cohort included 628 admissions (298 individuals). All patients in Scotland have a unique patient identification number (Community Health Index), which was used to obtain mortality data (date, cause and location) from National Records Scotland. Data collection continued until the end of 2014, resulting in a median mortality follow-up of 3.4 years (interquartile range [IQR] 2.3–5.3 years). Where patients had more than one DKA admission, data from their last admission was included for analysis. Mortality data are presented for the entire cohort identified by hospital admissions during the study period. Further analysis, investigating the influence of the number of lifetime DKA admissions, was limited to patients diagnosed with diabetes from 1981 onwards (when national data collection began). This population was stratified into three groups: one lifetime DKA episode, two to five lifetime DKA episodes and more than five DKA episodes, based on preliminary analysis of Kaplan–Meier survival curves.

All lifetime admissions (all Scottish hospitals) for DKA were ascertained from Scottish Morbidity Record (SMR01) coding from the Information Statistics Division of NHS National Services, Scotland [5], and was limited to those patients diagnosed after 1981 (when SMR01 data collection began). Similarly, any hospital admissions for the treatment of mental illness were recorded using SMR04 coding from the Information Statistics Division. National primary-care-level prescribing data have been collected in Scotland since 2009 (Prescribing Information System), and these data were interrogated to establish whether patients had received antidepressants, anxiolytics, antihypertensive agents, antiplatelet therapy or statins.

### Statistics

The *χ*^2^ test was used for categorical univariate analysis and the Kruskal–Wallis test was used for univariate analysis of continuous data. Non-parametric tests were selected as transformation did not provide satisfactory normal distribution, as determined by Kolmogorov–Smirnov tests. Two-sided *p* values were used to assess group differences and 0.05 was used as the threshold for significance. Univariate survival analysis was carried out using logrank tests for categorical predictors and univariate Cox models for continuous predictors. Proportional hazards assumptions were assessed using log(–log) plots for categorical predictors in a Cox model with no obvious violations. The Cox model was tested for the presence of interactions and none were found to be significant. Multivariate variable selection was performed using backward stepwise regression. Bonferroni correction was applied to post hoc testing to adjust for multiple comparisons. All statistical analyses were performed using SPSS version 22 (IBM, Armonk, NY, USA). Data are shown as median (IQR), unless otherwise stated.

## Results

### Differences between single and multiple DKA episodes

Lifetime national DKA admission data were available for all patients diagnosed with diabetes from 1981 onwards (271 patients from the complete cohort of 298). These patients were stratified into three groups based on the total number of DKA admissions: single admission (*n* = 96), two to five admissions (*n* = 111) and more than five admissions (*n* = 64). Overall, 55% (*n* = 164) of individuals presenting with DKA were men and no difference in the sexes was noted between the three categories (26.0% of women had more than five DKA presentations compared with 21.7% of men, *p* = 0.186). Compared with patients with a single admission, those with multiple presentations had a longer duration of diabetes (median 12.8 [IQR 10.0–17.5] vs 7.6 [2.3–13.6] years, *p* < 0.001) and were diagnosed with diabetes at a younger age (14 [9–23] vs 24 [16–34] years, *p* < 0.001). Multiple DKA admissions were more common in patients with higher levels of social deprivation (SIMD rank 1825 [813–3346] vs 2723 [1559–4310], *p* = 0.005) and higher HbA_1c_ levels (103 [89–108] vs 79 [66–96] mmol/mol; 11.6% [10.3–12.0%] vs 9.4% [8.2–10.9%], *p* < 0.001). Comparisons of other clinical variables can be seen in electronic supplementary material (ESM) Table 1.

Overall, 13.1% (8/61) of patients with more than five DKA admissions had experienced an inpatient admission for psychiatric care, compared with 5.6% (6/108) and 4.3% (4/92) of those with two to five and a single DKA admission, respectively (*p* = 0.092). Furthermore, 47.5% (29/61) of those with more than five DKA admissions had received antidepressants, compared with 27.8% in the intermediate (30/108) and 12.6% (12/95) in the single-attender groups (*p* = 0.001). No significant relationships were observed with DKA admission frequency and the use of other classes of medication (ESM Table 2).

### Inpatient DKA mortality rate

Over 6 years, 628 DKA admissions were identified, involving 298 individuals with type 1 diabetes. The median age at presentation was 28 years (IQR 22–40 years). No deaths were identified during inpatient admissions for the management of DKA. To ensure our data capture was complete, every death of a patient with type 1 diabetes registered to our outpatient clinic (153 deaths) was reviewed across the study time period using our comprehensive diabetes register. This revealed a single inpatient death where DKA was reported as a contributory factor; the inpatient DKA mortality rate was therefore, at most, 0.16%.

### Subsequent mortality following hospital discharge (total cohort)

In patients with a prior DKA presentation, 44 deaths (14.8%) were observed during follow-up (median 4.9 [IQR 3.3–6.7] years) in 298 individuals. The median age of death was 45.9 years (IQR 30.8–58.0 years). Mortality rates were significantly associated with the number of DKA presentations during the 6-year study period, and were highest in those with more than four admissions (29.6% [8/27]), intermediate in those with two to four admissions (18.3% [15/82]) and lowest in those with a single admission (10.6% [20/189]) (*p* = 0.016).

A total of 19 of these 44 deaths were of uncertain cause, and were often unexpected and potentially attributable to acute metabolic decompensation. The median age of these patients was 31 years (range 20–63 years) (full cause-of-death data are presented in ESM Fig. 1). Overall, 52.3% (23/44) deaths occurred at home at a median age of 38 years (IQR 27.7–52.3 years), compared with a median age of 57.7 years (IQR 40.5–61.4 years) in those dying in hospital (*p* = 0.01). Ten deaths (eight at home) occurred within 2 months of the final DKA admission; all of these deaths were unanticipated.

A range of clinical factors were associated with increased mortality risk, including older age, longer diabetes duration, prior DKA requiring intensive care admission, psychological issues, neuropathy, previous cardiovascular disease, excess alcohol intake and a longer length of stay during the last hospital admission (Tables 1, 2). Prescription of an antidepressant, at any time since 2009 (when national data collection began, *n* = 264), was associated with a trend towards increased likelihood of death (HR 2.24, 95% CI 0.99, 5.12, *p* = 0.055). Social deprivation was not associated with mortality risk in this cohort (Table 1).

**Table 1:** Clinical and laboratory variables at last DKA admission, comparing survivors with patients who subsequently died

Variable	Alive (*n* = 254)	Deceased (*n* = 44)	*p* value
Age at last presentation, years	28 (21–42)	43 (30–55)	<0.001
Diabetes duration, years	10 (5–16)	13 (9–27)	0.004
HbA_1c_, mmol/mol	90 (74–107)	82 (71–97)	0.220
HbA_1c_, %	10.4 (8.9–11.9)	9.7 (8.6–11.0)	0.220
SIMD rank, out of 6505	2677 (1423–4019)	2695 (1349–4333)	0.969
Length of stay at last DKA admission, days	2 (1–3)	4 (2–9)	<0.001
Hydrogen ion, nmol/l	72 (56–104)	78 (55–109)	0.423
Lactate, mmol/l	3.3 (2.2–4.7)	3.2 (1.8–3.9)	0.258
Glucose, mmol/l	33.5 (24.7–43.5)	36.0 (28.3–55.3)	0.067
ALT, μkat/l	0.35 (0.25–0.55)	0.35 (0.25–0.96)	0.468
Urea, mmol/l	8.3 (6.2–11.7)	14.2 (8.3–19.8)	0.002
Creatinine, μmol/l	121 (94–158)	159 (110–242)	0.002
White cell count, ×10^9^/l	18.3 (11.6–25.9)	14.8 (10.2–19.8)	0.055

Data are medians (IQR) and were compared using the Mann–Whitney *U* test

ALT, alanine aminotransferase

**Table 2:** Categorical clinical variables, comparing survivors with patients who subsequently died

Variable	Total (*n* = 298)	Alive (*n* = 254)	Deceased (*n* = 44)	Incidence rate ratio (95% CI)	*p* value
Psychological issues	109 (36.6)	86 (33.9)	23 (52.3)	2.60 (1.33, 5.08)	0.005
Retinopathy	126 (42.3)	106 (41.7)	20 (45.5)	0.77 (0.39, 1.53)	0.459
Nephropathy	33 (11.1)	24 (9.4)	9 (20.5)	1.53 (0.59, 3.93)	0.383
Peripheral neuropathy	64 (21.5)	47 (18.5)	17 (38.6)	2.36 (1.15, 4.82)	0.018
Autonomic neuropathy	36 (12.1)	25 (9.8)	11 (25.0)	2.47 (1.03, 5.95)	0.044
Ischemic heart disease	13 (4.4)	8 (3.1)	5 (11.4)	3.07 (0.94, 10.02)	0.063
Stroke/PVD	15 (5.0)	9 (3.5)	6 (13.6)	3.83 (1.35, 10.85)	0.012
Excess alcohol intake	41 (13.8)	30 (11.8)	11 (25.0)	3.83 (1.88, 7.82)	<0.001
Prior DKA admission to ITU	38 (12.8)	28 (11.0)	10 (22.7)	2.28 (1.03, 5.01)	0.041

Data are *n* (%), unless otherwise stated

ITU, intensive therapy unit; PVD, peripheral vascular disease

### Influence of lifetime DKA admissions

**Fig. 1:**
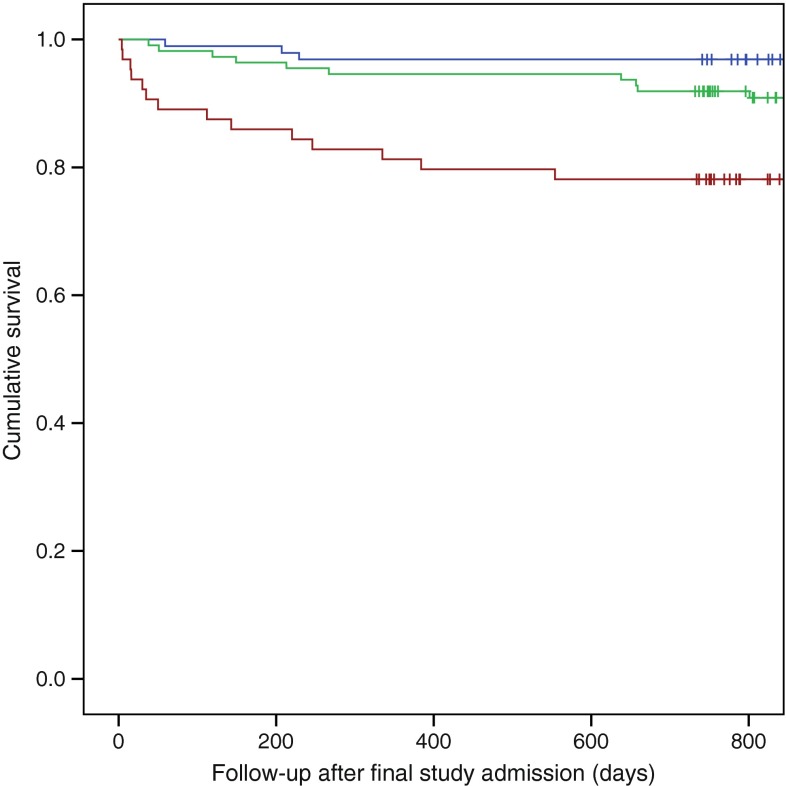
Kaplan–Meier curves stratified by lifetime DKA admissions. Blue line, single admission; green line, two to five admissions; red line, more than five admissions. Vertical lines indicate censored data

Overall, 23.4% (15/64) of those with more than five lifetime DKA admissions died over a median 2.4 year (IQR 2.0–3.8 years) follow-up period (HR 6.18 [95% CI 2.1, 18.3], *p* = 0.001; Fig. 1). Death occurred in 13.5% (15/111) of those with two to five admissions (HR 3.02 [95% CI 1.1, 8.4; reference is single DKA], *p* = 0.035) over a median of 3.7 years (IQR 2.4–5.5 years). A single lifetime DKA admission was associated with a 5.2% (5/96) risk of death during the follow-up period (median 4.1 [IQR 2.8–6.0] years). Median age at death was significantly lower in those with more than five DKA admissions (32 [IQR 23–39] years) than in those with two to five admissions (median 53 [IQR 40–58] years) or a single admission (median 53 [IQR 38–66] years) (*p* = 0.014). The prevalence of cardiovascular disease was not independently associated with mortality. Multivariate analysis identified greater number of DKA admissions, longer diabetes duration, previous psychiatric admissions and older age at diagnosis as independent predictors of death (Table 3).

**Table 3:** HRs for death following DKA, presented for all independently predictive variables from a multivariate Cox proportional hazard model

Variable	HR	95% CI for HR		*p* value
		Lower	Upper	
Diabetes duration (years)	1.069	1.018	1.121	0.007
Single DKA admission	Ref.	Ref.	Ref.	–
Two to five DKA admissions^a^	3.016	1.078	8.436	0.035
More than five DKA admissions^a^	6.176	2.089	18.256	0.001
Psychiatric inpatient admission	5.687	2.620	12.345	<0.001
Age at diagnosis	1.044	1.019	1.070	0.001

^a^For DKA frequency, single DKA admission is the reference

## Discussion

The most striking finding from this investigation is the substantial short-term risk of death associated with recurrent DKA admissions in patients with type 1 diabetes. Recurrent DKA disproportionately affects young people, typically those with greater social deprivation and markedly elevated HbA_1c_ levels. In keeping with other modern cohorts [2, 3], we found a low rate of inpatient mortality in patients presenting with DKA. Although recent evidence suggests suboptimal adherence to DKA management protocols in the UK [14], inpatient outcomes are almost always satisfactory. However, the frequency of subsequent sudden death at home, particularly in young people, is unacceptably high.

In the current study, we identified social deprivation as being significantly associated with an increased likelihood of multiple lifetime DKA presentations, as has previously been demonstrated in a cohort of 23,479 Scottish patients with type 1 diabetes, where the risk of admission for DKA was 4.5-fold higher for those in the most deprived quintile compared with the least deprived quintile, independently of other recognised risk factors such as HbA_1c_ [5]. This is broadly consistent with findings from the US T1D Exchange Clinic Registry, which identified lower income, lack of private medical insurance and lower educational attainment as independent risk factors for DKA admission [6]. Data from the T1D Exchange Clinic Registry also confirm a progressive increase in the likelihood of DKA with increasing HbA_1c_ [6]. Younger age at diagnosis of diabetes, and particularly during the teenage years, appears to be associated with an increased risk of multiple DKA presentations. Case series describing the experiences of patients with ‘brittle diabetes’ have recognised the potential impact of adjusting to a diagnosis of diabetes during adolescence [12]. In those diagnosed at a younger age, longer duration of diabetes does not account for the higher frequency of DKA presentations.

In the current study, almost 50% of the most frequent DKA attenders had received an antidepressant since 2009 and, although the absolute numbers were low, there was a trend towards a greater likelihood of admission for the treatment of psychiatric illness. Prior antidepressant prescription was associated with a higher risk of death and previous psychiatric inpatient care was independently predictive of death. The nationwide German/Austrian Diabetes Survey recently reported higher DKA rates in patients receiving antipsychotic medication [15] and, in a US series, depression and substance abuse were more common in those with multiple DKA presentations [16].

Most strikingly, a greater than one in five risk of death was observed in those with the highest frequency of DKA presentation over a median 2.4 years of follow-up, compared with a one in 20 risk of death in those with a single DKA admission over a median of 4 years. This represents a substantially elevated risk of death when compared with the Scottish type 1 diabetes population. Recently published national data reported mortality rates of 3.5 and 4.3 per 1000 person-years in men and women with type 1 diabetes, respectively, in the 30–34 years age bracket [4]. We identified frequency of DKA as an independent risk factor for death in multivariate analysis whilst, perhaps surprisingly, prior admissions for cardiovascular disease was not. This is potentially a reflection of the relatively low proportion of deaths due to cardiovascular causes; which might also explain why no significant differences were noted in social deprivation scores between those who died and survivors. The prevalence of prior cardiovascular disease was relatively low in this population, which therefore might have been underpowered to detect a significant difference. Modest associations were identified between mortality and neuropathy and nephropathy, but not retinopathy. This might reflect the high prevalence of retinopathy across both groups and failure to account for the broad spectrum of severity of this condition, from background changes to sight-threatening disease.

### Limitations

Whilst including two separate analyses (the complete cohort and a subset diagnosed with diabetes after 1981) might appear unnecessarily complicated, we chose to present both as the restricted dataset is strengthened by comprehensive national data on lifetime DKA admissions. However, solely limiting our analysis to these patients would have excluded those diagnosed with diabetes prior to the onset of national data collection in 1981, and would have distorted the total mortality figures. It is recognised that studies reliant on national data collection often under-report diabetes-related admissions [17]; this pitfall was avoided here through a combination of hospital-level and national data collection.

A significant benefit of the single-centre study design was our ability to carefully verify the quality of the included data, although reporting from a single centre does leave the generalisability of these results open to question. In defence of the wider applicability of these data, the characteristics of our patients seem broadly comparable with those described in several other series [6–8]. Despite a relatively small cohort size, we have been able to describe a number of significant associations in relation to the risks of both recurrent DKA and subsequent mortality.

As with any study reliant on death-certification data, recognition of the frequent lack of clarity in relation to ‘cause of death’ is necessary. The category we chose to describe as ‘unclear’ was the single largest cause of death and included unconfirmed hypoglycaemia and DKA (at home), as well as presumed sudden cardiac death. However, our most striking finding in relation to mortality was the high rate of death at home in very young patients, which was unequivocal.

Having recognised the significant mortality risk associated with recurrent DKA, it is important that these findings are verified, ideally through national registry data. Those diagnosed with diabetes in adolescence and those with mental health issues appear to be at particularly high risk for recurrent DKA, and efforts are required to develop effective support strategies. In the UK, attendance at a structured education programme that promotes flexible intensive insulin therapy (DAFNE) has been associated with a 61% reduction in DKA in the following 12 months [18]; however, it can be difficult to encourage those who have elected not to engage with specialist services to commit to a 5-day education programme. A potentially complementary strategy, targeting patients with persistently suboptimal HbA_1c_ levels (in type 1 and 2 diabetes), employs a combined medical, psychological and social support approach (‘3 Dimensions of Care for Diabetes’); this has been reported to result in significant reductions in emergency department attendances and inpatient admissions [19]. Longitudinal studies of ‘brittle diabetes’ suggest that patients eventually cease presenting with recurrent DKA, anecdotally often following positive life events, but frequently develop a significant burden of chronic diabetes complications [11, 12]. Considerable challenges remain in preventing early death and later complications in this vulnerable group.

## Electronic supplementary material

Below is the link to the electronic supplementary material.

ESM (PDF 260 kb)

## Abbreviations

DKA: Diabetic ketoacidosis
IQR: Interquartile range
SCI-Diabetes: Scottish Care Information–Diabetes Collaboration
SIMD: Scottish Index of Multiple Deprivation
