# Supplementary material for: Risk of death following admission to a UK hospital with diabetic ketoacidosis
Source: Diabetologia. 2016 Jul 11;59(10):2082–7. doi: 10.1007/s00125-016-4034-0 (PMC5016550; doi:10.1007/s00125-016-4034-0)

Electronic Supplementary Material

ESM table 1 : Comparison of clinical and biochemical features by frequency of lifetime DKA admission. Data are median (IQR) and are compared by Kruskal-Wallis test.

	Single admission (n = 96)	2 – 5 admissions (n = 111)	More than 5 admissions (n = 64)	P
Total admissions	1	3 (2 – 4)	10 (8 – 15)	<0.001
Diabetes duration at final follow-up admission (years)	7.6 (2.3 – 13.6)	9.5 (6.9 – 16.0)	12.8 (10.0 – 17.5)	<0.001
Age at final follow-up admission (years)	31 (23 – 42)	27 (20 – 43)	25 (22 – 36)	0.079
Age at diabetes diagnosis (years)	24 (16 – 34)	16 (12 – 29)	14 (9 – 23)	<0.001
Length of stay at last DKA admission (days)	2 (1 – 3)	2 (1 – 4)	2 (2 – 4)	0.367
SIMD rank	2723 (1559 – 4310)	3023 (1366 – 4288)	1825 (813 – 3346)	0.005
Cardiovascular disease present prior to inclusion in study (%)	7.3	13.5	7.8	0.265
Cardiovascular disease present at last DKA admission (%)	7.3	15.3	15.6	0.154
HbA1c (mmol/mol) [%]	79 (66 – 96) [9.4 (8.2 – 10.9)]	92 (76 – 115) [10.6 (9.1 – 12.7]	103 (89 – 108) [11.6 (10.3 – 12.0)]	<0.001
Hydrogen ion at last DKA (nmol/L)	72 (57 – 109)	72 (54 – 103)	77 (57 – 104)	0.767
Lactate at last DKA (mmol/L)	3.2 (2.1 – 5.1)	3.2 (2.2 – 4.3)	3.4 (2.2 – 4.6)	0.813
Glucose at last DKA (mmol/L)	35.1 (26.0 – 44.1)	28.9 (22.2 – 38.9)	36.0 (27.3 – 44.3)	0.005
Urea at last DKA (mmol/L)	9.1 (6.5 – 12.9)	8.4 (6.1 – 11.6)	8.2 (6.3 – 11.4)	0.474
Creatinine at last DKA admission (µmol/L)	134 (102 – 189)	119 (95 – 156)	118 (89 – 148)	0.036
White cell count at last DKA ($x10^9$/L)	19.4 (13.1 – 27.6)	18.3 (11.8 – 24.4)	15.4 (10.9 – 22.4)	0.098

ESM table 2: Differences in prescriptions of drug classes by frequency of DKA admission. Data are compared by Kruskal-Wallis test.

	Single admission	2 - 5 admissions	>5 admissions	p
Alive at beginning of 2009 (n)	95	108	61	
Prescribed antidepressants	12 (12.6%)	30 (27.8%)	29 (47.5%)	0.001
Prescribed antiplatelets	2 (2.1%)	9 (8.3%)	3 (4.9%)	0.236
Prescribed blood pressure medication	13 (13.7%)	18 (16.7%)	15 (24.6%)	0.615
Prescribed statins	12 (12.6%)	18 (16.7%)	8 (13.1%)	0.65
Prescribed anxiolytics	13 (13.7%)	7 (6.5%)	7 (11.5%)	0.054

ESM figure 1: Primary cause of death in patients with previous DKA admission.

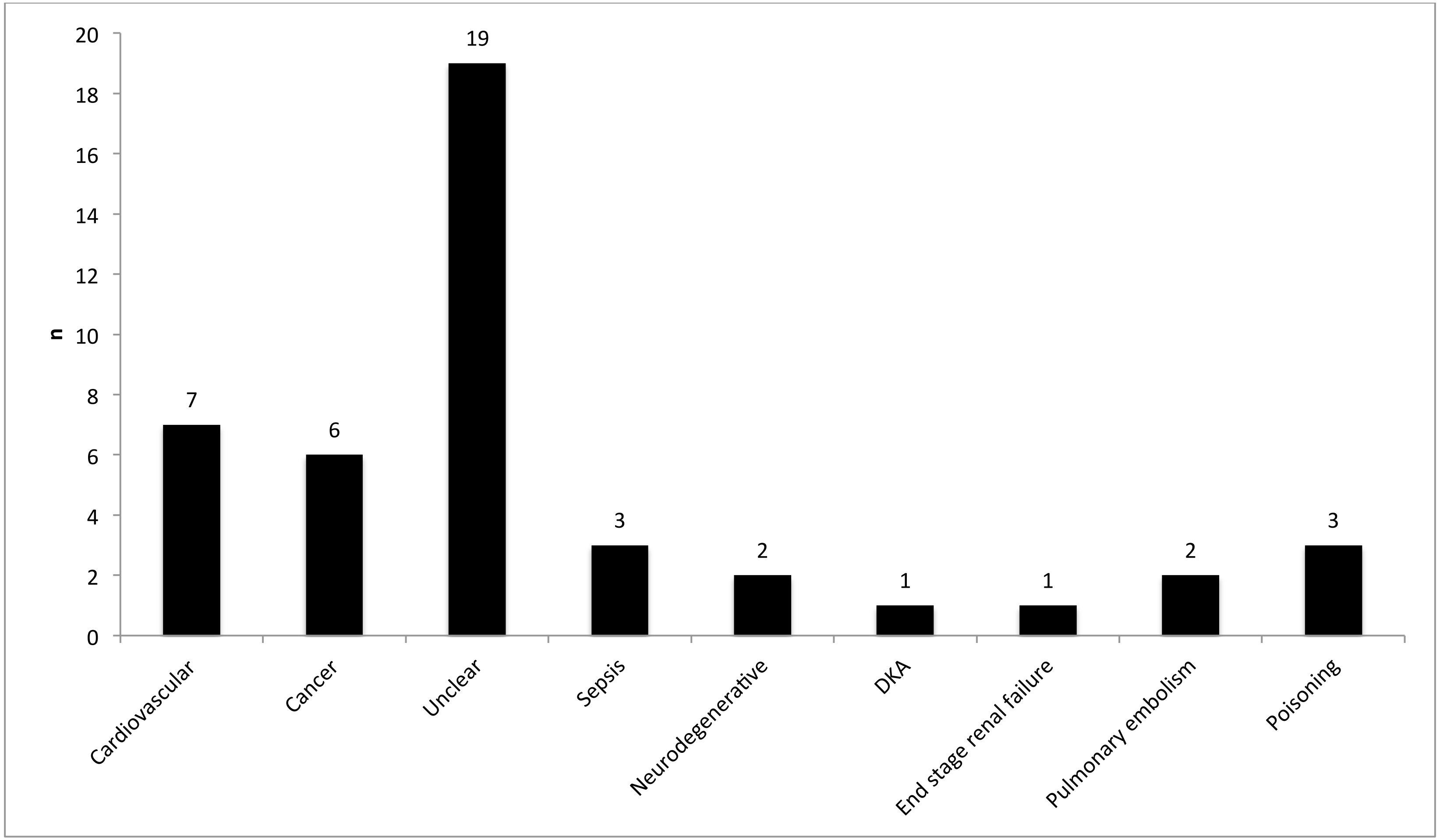

Supplement: Supplementary file 1 — (PDF 260 kb) [file 125_2016_4034_MOESM1_ESM.pdf]
